# Quality of Life in Saudi Vitiligo Patients

**DOI:** 10.4103/0974-2077.79188

**Published:** 2011

**Authors:** Luluah Al-Mubarak, Hind Al-Mohanna, Ahmed Al-Issa, Monzer Jabak, Sanjeev V Mulekar

**Affiliations:** *College of Medicine, King Saud University, Riyadh, Kingdom of Saudi Arabia*; 1 *National Center for Vitiligo, Riyadh, Kingdom of Saudi Arabia*; 2 *Derma Clinic, Riyadh, Saudi Arabia*

**Keywords:** Vitiligo, quality of life, culture, ethnicity, Saudi Arabia, relationships, self-respect

## Abstract

**Background:**

Vitiligo has a devastating psychosocial effect. The cultural traditions of Saudi society are quite different compared with the western world. Hence, a quality of life study using a different questionnaire suitable to the cultural traditions of the society is necessary to measure qualify of life in vitiligo patients.

**Objective:**

This study was conducted to assess the quality of life (QOL) in Saudi vitiligo patients and their family.

**Materials and Methods:**

A prospective cross-sectional study at National Center for Vitiligo and Psoriasis, Saudi Arabia. A validated Arabic questionnaire of 41 questions was developed and utilized specifically for this study. Arabic language instrument was distributed to 260 vitiligo patients. Scores were compared in relation to demographic, clinical, and social variables in 4 dimensions of scale (relationship with colleagues, family relationship, social relationship, and self respect).

**Results:**

Overall score QOL was 17.1. Mean score for males was 11.1, whereas that for females was 23.9 (*P* < 0.05). Females scored significantly higher in all the 4 dimensions. Patients with exposed disease lesions scored significantly higher than those with unexposed lesions 5 vs 3.4 (*P* < 0.05).

**Conclusion:**

The overall score of QOL in vitiligo is relatively high, indicating a negative impact of the disease on QOL. QOL in women is significantly more affected than in men.

## INTRODUCTION

Vitiligo is an acquired skin disorder characterized by loss of melanocytes in the epidermis. Vitiligo has been confused with other diseases with similar clinical features, such as leprosy, which has increased the stigma.

In the Kingdom of Saudi Arabia, vitiligo plays an important role not only in the lives of affected individuals, but also their families and social circles. Also, the majority of population have skin type IV and V, and hence vitiligo is associated with negative impacts.

It is associated with low self-esteem, tendency to social isolation, and poor quality of life.[1] Hence the management of vitiligo should include the biopsychosocial approach in order to achieve full satisfaction of the patients. Disturbance of life aspects severely affect the outcome of medical interventions.

Quality of life (QOL) is currently a scientifically measurable tool that can be validated. In dermatology, the 3 most important and widespread QOL tools are the Skindex,[2] the Dermatology Life Quality Index (DLQI),[3] and the Dermatology-Specific Quality of Life Questionnaire.[4] A children’s DLQI was also introduced.[5] A cartoon version using the same validated questions was developed in full color.[6]

QOL scales can be used to compare disability between different skin disorders or other diseases. This enables researchers to better assess the implications of diseases and to measure the efficacy of medications and treatment.[1,7]

Most studies on QOL in skin disorders in general and vitiligo in particular, have stressed the impact on psychological, social, emotional, and economic status of affected patients.

The current study is aimed at studying QOL in vitiligo patients in order to assess its impact on them, their family, and social life.

## MATERIALS AND METHODS

This study was conducted from July to December 2006. A questionnaire was developed, which consisted of 92 questions. It was further shortened to 41 questions.

Validity and reliability tests were done on 26 candidates. The 41 questions were grouped into 4 dimensions. 1: Relation with colleagues (11) questions; 2: family life (11) questions; 3: social relationships (9) questions; and 4: self-respect (10) questions. Demographic and clinical information were also included in the questionnaire.

The questionnaire was distributed to vitiligo patients after explaining the purpose of the research and the method of filling the questionnaire. The time required to complete the questionnaire was 12–15 min.

Data were analyzed using the SPSS software package. The inclusion criteria were Saudis over 18 years of age with vitiligo, newly diagnosed within last 6 months.

All other Saudi patients presented to the clinic were included during the duration of the study. The tools used to make an analysis include dependent and independent t test, ANOVA test, and Scheffe’s test.

## RESULTS

Table 1 illustrates the age and sex distribution of the study subjects. The mean age for males and females was 33 and 31 years, respectively. Age range for males was 19–64 years (SD 9.8), while that for females was 18–53 years (SD 7.6). No statistically significant difference in age between the 2 groups was noted (*P* > 0.05).

**Table 1:** Age and sex distribution among study subjects

Age (years)	Male (%)	Female (%)	Total (%)
18–20	3 (2)	5 (4)	8 (3)
21–30	72 (51)	65 (54)	137 (53)
31–40	34 (24)	40 (33)	74 (28)
41–50	20 (14)	8 (7)	28 (11)
51–60	8 (6)	2 (2)	10 (4)
>60	3 (2)	—	3 (1)
Total	140 (100)	120 (100)	260 (100)

In the male group, 39% of males were single, 60% were married, and 1% were divorced. In the female group, 33% were single, 58% were married, and 8% were divorced.

Table 2 depicts the educational status for study subjects by gender. About 37% of females and males had secondary education or less. However, 14% of males had higher than college degree education as compared with less than 1% of females.

**Table 2:** Educational status of study subjects by gender

Educational status	Male (%)	Female (%)	Total (%)
Illiterate	—	4 (3)	4 (2)
Reads and writes	1 (1)	2 (2)	3 (1)
Primary	1 (1)	5 (4)	6 (2)
Intermediate	11 (8)	6 (5)	17 (7)
Secondary	40 (29)	27 (22)	67 (26)
College	63 (45)	71 (59)	134 (52)
Masters	19 (14)	—	19 (7)
Doctorate	1 (1)	1 (1)	2 (1)
Other	4 (3)	4 (3)	8 (3)
	140 (100)	120 (100)	260 (100)

The minimum score in the 4 dimensions was zero. The maximum score was 33 in each of the relationship with colleagues and family relationship dimensions, whereas that in each of social relationship and self-respect was 27 and 30, respectively.

Table 3 demonstrates these differences in each dimension and in the total score of the scale. The total score for males on the QOL scale was 11.1 as compared with 23.92 in females. This difference is statistically significant (*P* < 0.01) and denotes that impact of QOL in female patients with vitiligo is significantly more affected than their male counterparts. The table also shows that there are statistically significant differences in mean scores of QOL between males and females in all 4 dimensions of the scale. Males have less mean scores than females, which means that vitiligo in males does not cause them as much problems with their colleagues, their family life, social relationships, and self-respect as is the case with females who scored significantly higher.

**Table 3:** Quality of life scores in study subjects by gender

	Relationship with colleagues	Family relationship	Social relationship	Self-respect	Total score
Male(mean, SD)	(2.0, 3.5)	(2.8, 4.1)	(2.7, 4.4)	(3.6, 4.8)	(11.1, 14.6)
Female (mean, SD)	(2.4, 4.6)	(5.7, 6.2)	(6.0, 6.4)	(8.8, 7.8)	(23.9, 21.7)
*t* value significance	2.8*	4.5*	5.0 *	6.5*	5.7*

* Significant at 0.01

Table 4 shows that there are statistically significant differences (*P* < 0.01) in family life and social relationship dimensions. It also shows that there are statistically significant differences at the 0.05 level in the self-respect, social status dimension, and the total score. The Scheffe’s test showed that married subjects had less impact on QOL (mean score, 3.24) as compared with single subjects (mean score, 5.08). A similar difference in the same dimension was noted between married and divorced subjects. Married subjects scored 3.24, whereas divorced subjects scored 7.75 (*P* < 0.05).

**Table 4:** Quality of life scores in study subjects by social status (single, separated, married, widow, divorced, and others)

	Relationship with colleagues	Family relationship	Social relationship	Self- respect	Total score
Between groups					
Sum of squares	62.2	364.8	28.9	287.3	2236.8
Degree of freedom	2	2	2	2	2
Mean of squares	31.1	182.4	14.4	143.6	1118.4
Within group					
Sum of squares	4213.2	7021.9	8241.3	11900.7	93918.5
Degree of freedom	257	257	257	257	257
Mean of squares	16.4	27.3	32.0	46.3	365.4
*F* value	1.9	6.7**	0.5	3.1*	3.1*
Significance					

* Significant at 0.05

** Significant at 0.01

Table 5 demonstrates differences in scores of QOL scale in study groups depending on their occupation. The t test showed significant differences (*P* < 0.05) in the total QOL score and the self-respect dimension. The Scheffe’s test showed significant differences (*P* < 0.05) between teachers and each of the administrative employees, merchants, retired persons, and soldiers. The average QOL score for each of the retired, merchant, government employee, soldier, other, and teacher were 2.2, 2.5, 3.7, 4.5, 7.3, and 7.7 respectively. In retired persons, lowest QOL scores were clearly shown (less QOL impact), whereas teachers scored highest (higher QOL impact). No significant differences in QOL scores were noted in each of the relationship with colleagues, family relationships, and social relationship dimensions.

**Table 5:** Quality of life scores in study subjects occupation (teacher, employee, retired, nurse, physician or pharmacist, soldier, businessman, and others)

	Relationship with colleagues	Family relationship	Social relationship	Self- respect	Total score
Between groups					
Sum of squares	106.8	320.3	297.1	886.6	5392.5
Degree of freedom	7	7	7	7	7
Mean of squares	15.3	45.7	42.4	126.6	770.4
Within group					
Sum of squares	3627.7	5967.9	6812.6	9777.6	76342.2
Degree of freedom	227	227	227	227	227
Mean of squares	16.0	26.3	30.0	43.1	336.3
*F* value	1.0	1.7	1.4	2.9 *	2.3 *
Significance					

* Significant at 0.05

Table 6 depicts differences of QOL scores in study subjects based on the educational level of these subjects. ANOVA test showed significant differences (*P* < 0.05) only in the social relationship dimension. The Scheffe’s test showed that Masters Degree holders had lowest scores compared with all others (QOL mean score of 1.26 as compared with 10.7, 9.7, and 5.6 for illiterates, read and write, and intermediate education, respectively). The test also showed no significant differences among subjects in the relationship with colleagues, family relationship, and self-respect dimensions.

**Table 6:** QOL scores in study subjects by education (illiterate, reads and writes, primary, intermediate, secondary, college, masters, doctorate and others)

	Relationship With colleagues	Family relationship	Social relationship	Self respect	Total score
Between groups					
Sum of squares	211.1	101.0	485.9	455.8	3916.3
Degree of freedom	8	8	8	8	8
Mean of squares	26.4	12.6	60.7	57.0	849.5
Within group					
Sum of squares	4064.4	7285.5	7784.2	11732.1	92239.4
Degree of freedom	251	251	251	251	251
Mean of squares	16.2	29.0	31.0	46.7	367.5
*F* value	1.6	0.4	1.9 *	1.2	1.3
Significance					

* Significant at 0.05

Table 7 illustrates differences in QOL scale score in the study groups in relation to their age groups. ANOVA test showed significant differences (*P* < 0.01) in each of the relationships with colleagues, family relationship, self-respect, and the total scale scores. The Scheffe’s test showed significant differences (*P* < 0.05) between the 21–30 years age group and each of 31–40, 41–40, 51–60 age groups. This age group scored highest in all dimensions (3.4, 5.3, 7.4, and 21.2, respectively) for each of the relationship with colleagues, family relationship, self-respect, and total score, respectively, which show more impact on QOL in the younger group. No significant differences related to age were noted in the social relationship dimension.

**Table 7:** QOL scores in study subjects by age groups

	Relationship with colleagues	Family relationship	Social relationship	Self respect	Total score
Between groups					
Sum of squares	248.7	434.0	283.5	687.3	6045.5
Degree of freedom	5	5	5	5	5
Mean of squares	49.7	86.8	56.7	137.4	1209.1
Within group					
Sum of squares	4026.8	6952.5	7986.6	11500.7	90110.2
Degree of freedom	254	254	254	254	254
Mean of squares	15.8	27.4	31.4	45.3	354.8
*F* value	3.1_**_	3.2_**_	1.8	2.9 *	3.0_**_
Significance					

* Significant at 0.05

* Significant at 0.01

Table 8 shows differences in scores of the QOL scale in study groups in relationship to location of disease lesions. Lesions were divided into “exposed” (face, neck, hand, fingers, and palms) vs “unexposed” lesions (rest of the areas, ie, abdomen, chest, back, genital areas, legs, and others). Independent t test clarify that only the social relationship dimension showed significant differences (*P* < 0.05) between the exposed (mean score, 5.0) and the nonexposed (mean score, 3.4). However, the same group scored higher in all other dimensions and the total scale, although such difference was not significant (15.5 vs 19).

**Table 8:** QOL scores in study by disease exposure

Dimension		No.	Mean scores	S.D.	T value significance
Relationship	Non-exposed	144	2.4	3.8	1.2
with colleagues	Exposed	116	3.0	4.3	
Family	Non-exposed	144	3.7	5.3	1.4
relationship	Exposed	116	4.6	5.4	
Social	Non-exposed	144	3.6	5.3	2.1 *
relationship	Exposed	116	5.0	5.9	
Self	Non-exposed	144	5.8	7.1	0.5
respect	Exposed	116	6.3	6.6	
Total	Non-exposed	144	15.5	18.7	1.4
score	Exposed	116	19.0	19.8	

* Significant at 0.05

## DISCUSSION

In Asian population with darker skin colour, vitiligo is associated with major social, emotional, economic, and psychological negative implications for patients with the disease and their families. It is believed that better understanding of QOL components and the degree of its impact on patients can lead to better management and ways to handle this disease.

Although there are well-known validated instruments dealing with dermatological diseases and their impact on QOL, we preferred to establish another questionnaire. The need behind creation of new scale is that Arabs, especially population in gulf countries, have distinctive social, religious, and cultural structure. This will allow Arab researchers to use it in different countries and enable them to compare results with others.

The questionnaire contained 4 dimensions related to the family life, life with colleagues at school and work, their wider circle of social life, and the way they look inside their own selves and reflect how they truly feel about being effected with vitiligo.

Our study showed that vitiligo has a major impact on QOL of female subjects compared with male subjects. The total scale mean score on the questionnaire for males was 11.1, whereas that for females was 23.9. This difference was statistically significant *P* < 0.01. Due to the nature and length of our questionnaire, it cannot be accurately compared with other scales but clearly goes along with the DLQI of Finlay and others.

Gender can play a crucial role in the impairment of quality of life. Borimenjad *et al*,[8] and Cotterill *et al*,[9] showed that females had significantly more overall impairment of QOL as compared with males.

Vitiligo can be localized to hidden or exposed body areas but can also spread to cover major surfaces or the whole body surface. Shah and Coats[10] (2005), Beattie and Lewis-Jones,[11] reported that dermatology QOL is not affected by age in their study group but is significantly affected by extent of skin disease.

Cotterille and Cunliffe[9] and other investigators have shown that girls generally experience more psychological morbidity than boys. Our study did show a clear and significant higher score in females as compared with males on the total scores and in every dimension of the score. This can be attributed to the fact that females may feel less attractive and are more concerned of lower chances to get married.

Marital status showed differences in effects in the total score of the scale and 2 of its dimensions: the “family relationship” and the “self-respect.” Married subjects have less impact on their QOL as compared with single and divorced subjects. This could be explained by the more secured and stable life married subjects enjoy.

Our study shows a statistically significant difference in total scale scores and in the “self-respect” dimension among different occupations. Teachers scored very high compared with other occupations. This could be because teachers are always exposed to their class students and are under continuous focus. Retired individuals scored lowest probably because they are already old, have got used to the disease and with stable life. Educational level of vitiligo patients may play an important role in understanding and management of their own disease. Our study showed that those with a higher education “Masters degree” and above have lowest scores compared with all other subjects, which were only significant in the “social relationship” dimension probably due to the fact that this dimension is the one that has most interaction with others outside the nuclear family boundaries. Disease spread and extension to extremities, face, and neck are thought to have special impact on subjects affected with vitiligo. The finding reported by Shah and Coats[10] that dermatology QOL is affected by extent of skin disease was not strongly demonstrated in our study except in the “social relationship” dimension where those with exposed disease lesions scored significantly higher than those with unexposed lesions. This could be because patients’ families and colleagues in work get used to unusual appearance of the skin lesions and patients themselves have the same feeling towards exposed and nonexposed depigmented areas.

## CONCLUSION

Females have been shown to be more effected by vitiligo in all dimensions of the scale. It is emphasized that efforts towards management should consider the psychosocial and economic factors, especially in females, in addition to therapeutic efforts.
